# Dr. Alfred J. Bigelow

**Published:** 1909-03

**Authors:** 


Dr. Alfred J. Bigelow, of New York, a graduate of the University of Buffalo, Medical Department, 1851, died at his home January 22, 1909, of senile debility, aged 73 years.

